# Cardiovascular risk assessment using the lipid accumulation product index among primary healthcare users: a cross-sectional study

**DOI:** 10.1590/1516-3180.2018.0293240119

**Published:** 2019-07-15

**Authors:** Jallyne Nunes Vieira, Marina Augusta Dias Braz, Flayane Oliveira Gomes, Priscilla Rafaella da Silva, Ohanna Thays de Medeiros Santos, Ilanna Marques Gomes da Rocha, Iasmin Matias de Sousa, Ana Paula Trussardi Fayh

**Affiliations:** I Undergraduate Student at Health Science College of Trairi, Universidade Federal do Rio Grande do Norte (UFRN), Santa Cruz (RN), Brazil.; II Undergraduate Student at Health Science College of Trairi, Universidade Federal do Rio Grande do Norte (UFRN), Santa Cruz, RN, Brazil.; III Undergraduate Student at Health Science College of Trairi, Universidade Federal do Rio Grande do Norte (UFRN), Santa Cruz (RN), Brazil.; IV Undergraduate Student at Health Science College of Trairi, Universidade Federal do Rio Grande do Norte (UFRN), Santa Cruz (RN), Brazil.; V Undergraduate Student at Health Science College of Trairi, Universidade Federal do Rio Grande do Norte (UFRN), Santa Cruz (RN), Brazil.; VI BSc. Master’s Degree Student in Postgraduate Program on Nutrition, Health Science Center, Universidade Federal do Rio Grande do Norte (UFRN), Natal (RN), Brazil.; VII BSc. Master’s Degree Student in Postgraduate Program on Nutrition, Health Science Center, Universidade Federal do Rio Grande do Norte (UFRN), Natal (RN), Brazil.; VIII PhD. Professor of Postgraduate Program on Nutrition, Health Science Center, Universidade Federal do Rio Grande do Norte (UFRN), Natal (RN), Brazil.

**Keywords:** Obesity, Overweight, Cardiovascular diseases, Primary health care, Waist circumference

## Abstract

**BACKGROUND:**

The lipid accumulation product (LAP) index is an abdominal adiposity marker.

**OBJECTIVE:**

The aim of this study was to describe the cardiovascular risk of primary healthcare users through the LAP index and correlate it with anthropometric and biochemical indicators.

**DESIGN AND SETTING:**

Cross-sectional study in primary care units in a city in northeastern Brazil.

**METHODS:**

The subjects responded to a structured questionnaire that contained questions about their sociodemographic condition, and then underwent an anthropometric nutritional assessment. The LAP index values were expressed as three degrees of cardiovascular risk intensity: high risk (above the 75^th^ percentile), moderate risk (between the 25^th^ and 75^th^ percentiles) and low risk (below the 25^th^ percentile).

**RESULTS:**

The median LAP index was 52.5 cm.mmol/l (range: 28.2-86.6), and there was no statistically significant difference between the sexes: 57.7 cm.mmol/l (24.5-91.1) and 49.5 cm.mmol/l (29.8-85.2) for females and males, respectively (P = 0.576). Among all the subjects, 67.2% were overweight and there was a statistically significant difference in mean LAP index between those who were and those who were not overweight. Statistically significant differences in anthropometric and biochemical markers for cardiovascular risk were observed among individuals who had higher LAP index values. There were significant correlations between the LAP index and all of the biochemical variables.

**CONCLUSIONS:**

These significant correlations between the LAP index and the traditional biochemical risk markers may be useful within conventional clinical practice, for cardiovascular risk screening in primary healthcare.

## INTRODUCTION

Cardiovascular diseases (CVD) are considered to be one of the most serious public health problems because of their multidimensional nature and all the consequences that they have for the individuals affected, their families and the healthcare system.^1^ Obesity is becoming a global epidemic and, additionally, the prevalence of CVD risk is increasing. Therefore, it is important to know the magnitude of CVD risk factors in order to implement healthcare planning such that effective intervention in this reality becomes possible.^2^ Hence, to predict CVD risk, it is necessary to sum the factors and analyze how synergisms between them may influence the development of coronary events.^3^

Body mass index (BMI) is the recommended indicator for diagnosing and classifying obesity.^2^ However, it determines overall obesity and does not have the capacity to identify how body fat is distributed. Thus, it has limitations with regard to distinguishing excess fat and lean mass, and with regard to determining the anatomical locations or functions of different fat deposits, especially because fat accumulated in the abdominal region is more associated with metabolic diseases than is the BMI alone.^4^^,^^5^ Therefore, it is important to also use other cardiovascular risk indexes, in order to improve screening accuracy.

The lipid accumulation product (LAP) is one of these indexes. It is based on a combination of two safe and inexpensive measurements: waist circumference and serum triglycerides.^6^ These tend to increase with age, which suggests that overaccumulation of lipids occurs over time.^7^ The LAP index may thus constitute an abdominal adiposity marker that correlates with central lipid accumulation.^8^

Since the LAP index was first proposed, in Kahn’s pioneering study,^9^ many researchers have used it to predict cardiovascular risk in different populations, such as among patients with type 2 diabetes^6^ or insulin resistance,^10^ women with polycystic ovarian syndrome^10^^,^^11^ and hospital outpatients^12^ and inpatients.^7^ However, no studies have evaluated the use of this index among patients attending primary care units.

While all adults and elderly people should have their cardiovascular risk assessed, this is not practical in many developing countries. In such countries, decision-making about whom to screen and when to do this is resource-dependent.

According to the European guidelines on cardiovascular disease prevention in clinical practice,^13^ opportunistic screening for cardiovascular risk is a form of screening that is done when the opportunity arises. For example, such opportunities may arise when individuals attend consultations with their general practitioners for some other reason, as occurs in primary care units. In Brazil, the aim in primary care units is to resolve up to 80% of the population’s health problems, without the need for referral to hospitals.

## OBJECTIVE

Thus, the objective of this study was to describe the CVD risk of primary healthcare users through the LAP index and correlate this with anthropometric and biochemical indicators for cardiovascular risk.

## METHODS

### Study population and sampling procedure

This was a cross-sectional study on a population of adults and elderly people of both sexes who were living in a city in northeastern Brazil and attending primary healthcare units. The exclusion criteria of the study were that the subjects should not be pregnant women and should not be adults or elderly people with the any of the following conditions: liver disease, ascites, acquired immune deficiency syndrome, malnutrition or renal replacement therapy.

During the data collection period, the researchers invited all users who were present at the healthcare unit and who met the inclusion criteria to participate in the study. After these individuals had voluntarily agreed to participate in the study, they signed an informed consent statement in duplicate.

This research project had previously been approved by the ethics committee of the Federal University of Rio Grande do Norte (Universidade Federal do Rio Grande do Norte, UFRN), under protocol no. 284,437 and ethics committee no. CAAE 13148313.1.0000.5568, on May 26, 2013. All the procedures used were in accordance with the ethical standards of the committee responsible for human experimentation and with the Helsinki Declaration.

The total population of the city and the prevalence of high LAP index that was found in a previous study in Brazil^4^ were taken into consideration in calculating the sample size. The study power was set at 80% and the significance level at 5%. The required minimum number of patients was found to be 384 individuals.

### Data collection

Data collection took place between July 2014 and October 2015, during the mornings. Firstly, the patients answered a structured questionnaire that asked for information about their housing, socioeconomic condition and lifestyle. Their economic class was evaluated using the ABEP (Brazilian Association of Polling Companies) methodology,^14^ which takes into account the numbers of material goods and monthly-paid employees in the household and the educational level of the main income earner. From this, the subjects were clustered into economic classes ­A1/­A2, B1/B2, C1/C2, D and E, which were subsequently regrouped into: A/B (better condition), C (intermediate condition) and D/E (worse condition). Economic classes D and E are predominant, according to the ABEP.

Body mass measurements were obtained using a properly calibrated professional mechanical scale with stadiometer (model 110 CH, Welmy, Brazil) with a capacity of up to 150 kg. The subjects were asked to remove their shoes and heavy clothing, such that they were then weighed while only wearing minimal clothing. For height measurements, the subjects stood barefoot at the center of the equipment, with their feet together and arms extended down the sides of the body, and the Frankfurt horizontal plan was used. BMI was then calculated and nutritional status was then ranked in accordance with the cutoffs recommended by the World Health Organization (WHO).^2^

Waist circumference (WC) was measured using an inelastic tape at the midpoint between the lower costal margin and the iliac crest. The cutoff for excess abdominal adiposity was set at 102 cm for men and 88 cm for women, as recommended by the National Cholesterol Education Program (NCEP/ATP III, 2001).^15^ Hip circumference was measured with the measuring tape positioned at the point of greatest circumference in the gluteal region. The waist-to-hip ratio (WHR) was then calculated by dividing waist circumference by hip circumference. The WHR cutoff points established by WHO^2^ were implemented, such that values of > 1 and > 0.85 represented the risk of developing cardiovascular disease among men and women, respectively.

The biochemical test results were collected from the patients’ records. Results from tests that had been performed within 30 days prior to the anthropometric evaluation were accepted. Total cholesterol and fractions, triglycerides and blood glucose, all measured in a fasting state, were recorded. The LAP index was determined through the equation proposed by Kahn,^9^ according to sex. For women, this was defined as [(waist circumference in cm - 58) x (triglycerides in mmol/l)]; while for men, this was [(waist circumference in cm - 65) + (triglycerides in mmol/l)]. High cardiovascular risk was ranked according to the LAP index, such that the 75^th^ percentile (P75) was taken to be the cutoff for data distribution. Triglyceride measurements in mg/dl were converted to mmol/l.

### Statistical analysis

The data were organized in the Microsoft Excel software and were then exported to the Statistical Package for the Social Sciences (SPSS) software version 20.0 for Windows, for analysis. The normality of the distribution of the quantitative variables was tested using the Shapiro-Wilk test. LAP index values were expressed in quartiles using three degrees of cardiovascular risk intensity: high risk (above the 75^th^ percentile), moderate (between the 25^th^ and 75^th^ percentiles) and low (below the 25^th^ percentile). The categorical variables were correlated using the chi-square test, and quantitative variables were compared using the t test for independent samples, or the Mann-Whitney test for nonparametric data. Correlations were tested using the Pearson correlation test. P ≤ 0.05 was considered significant for all analyses.

## RESULTS

The subjects were aged 18-90 years, and their mean age was 48.4 ± 15.9 years. Table 1 shows the description of the sample data. Predominance of females and married, retired and sedentary individuals were observed. Sedentarism was defined as < 150 minutes of moderate-intensity physical activity per week. The subjects’ economic class was consistent with populations that are attended through the public healthcare system, in that these subjects presented lower levels of financial resources (economic classes D and E).

**Table 1.** Sociodemographic description of the sample

Variable	n = 437	%
Sex		
Male	115	26.3%
Female	322	73.7%
Occupation		
Retired	93	21.3%
Housework (unemployed)	69	15.8%
Farmer	47	10.9%
Self-employed	37	8.5%
Teacher	11	2.5%
Domestic worker	19	4.3%
Other	155	35.5%
Marital status		
Married	275	62.9%
Single	118	27%
Widower	26	5.9%
Divorced	18	4.1%
Economic class		
A/B	0	0%
C	198	45.3%
D/E	239	54.7%
Active physically		
Yes	141	31.3%
No	296	67.7%
Smoker		
Yes	67	15.6%
No	312	71.4%
Former smoker	57	13%

The median LAP index in the general population was 52.5 ­cm.­mmol/l (range: 28.2-86.6), and there was no statistically significant difference between the sexes: 57.7 cm.mmol/l (24.5-91.1) and 49.5 cm.mmol/l (29.8-85.2) for females and males, respectively (P = 0.576). Among all the subjects, 293 were overweight (67.2%), and there was a statistically significant difference in mean LAP index between those who were and those who were not overweight: 99.03 ± 74.76 cm.mmol/l and 48.86 ± 50.60 cm.mmol/l, respectively (P < 0.01).

Table 2 provides an overview of cardiovascular risk prevalence using different anthropometric methods and the LAP index. From the data presented, it can be seen that these subjects showed significant cardiovascular risk. The indicator that pointed towards the highest cardiovascular risk for both sexes was the WHR, and this was also higher among men. Among women, the LAP index was the indicator that identified the highest prevalence of cardiovascular risk. In comparing the sexes, the prevalence of cardiovascular risk factors was only different between the sexes regarding increased WC and WHR.

**Table 2.** Comparison of prevalence of cardiovascular risk parameters between sexes

	Total	Male	Female	P-value*
Obesity	133 (30.5%)	38 (33%)	95 (29.6%)	0.834
High waist circumference	84 (19.2%)	42 (36.5%)	42 (13.0%)	< 0.01
High waist-to-hip ratio	202 (64.1%)	74 (84.1%)	128 (56.4%)	< 0.01
High lipid accumulation product index (P75)	109 (24.9%)	34 (31.2%)	75 (23.3%)	0.182

*P-values obtained using the chi-square test.

Table 3 summarizes the correlation analysis on the BMI, WHR and LAP index, in relation to the traditional biochemical parameters of cardiovascular disease. The LAP index showed statistically significant correlations with all the biochemical parameters analyzed. This was better than was seen regarding WHR and BMI, which showed significant correlations with only three and two biochemical variables, respectively. These results demonstrate that the LAP index presented higher power for screening individuals with metabolic abnormalities than did BMI. However, specifically in relation to glycemia, the WHR was the indicator that showed the strongest correlation. The strongest correlation for high-density lipoprotein (HDL)-cholesterol was with BMI.

**Table 3.** Correlations of body mass index, waist-to-hip ratio and lipid accumulation product (LAP) index with biochemical parameters

	Body mass index	Waist-to-hip ratio	LAP index
Glycemia	r = 0.106 P = 0.03	r = 0.249 P ≤ 0.01	r = 0.183 P ≤ 0.01
Total cholesterol	r = 0.013 P = 0.79	r = 0.112 P = 0.05	r = 0.248 P ≤ 0.01
HDL-cholesterol	r = -0.314 P ≤ 0.01	r = -0.295 P ≤ 0.01	r = -0.296 P ≤ 0.01
LDL-cholesterol	r = 0.040 P = 0.52	r = 0.096 P = 0.13	r = 0.151 P ≤ 0.01

P-values obtained using the Pearson correlation test.

HDL = high-density lipoprotein; LDL = low-density lipoprotein.

Lastly, Table 4 shows a comparison between biochemical test values and BMI among people who were classified as presenting high cardiovascular risk (≥ P75) and low cardiovascular risk (< P75) according to the LAP index. With the exception of total cholesterol and low-density lipoprotein (LDL) cholesterol, there were statistically significant differences in relation to all the variables tested, thus demonstrating that people with higher LAP index values also showed negative changes in other cardiovascular risk parameters.

**Table 4.** Comparison between biochemical test values and body mass index, in terms of high cardiovascular risk (above P75) and low cardiovascular risk (below P75) according to the lipid accumulation product (LAP) index

Variables	LAP percentile 75	Mean ± SD	P-value
Triglycerides (mg/dl)	Below P75	136.87 ± 56.36	< 0.01^a^
	Above P75	297.17 ± 146.50	
Total cholesterol (mg/dl)	Below P75	197.18 ± 45.29	0.19^b^
	Above P75	222.0 ± 54.575	
HDL-cholesterol (mg/dl)	Below P75	51.22 ± 13.98	< 0.01^b^
	Above P75	42.10 ± 10.03	
LDL-cholesterol (mg/dl)	Below P75	118.75 ± 39.61	0.17^a^
	Above P75	125.80 ± 42.84	
Glycemia (mg/dl)	Below P75	101.09 ± 38.37	< 0.01^a^
	Above P75	120.46 ± 57.35	
Body mass index (kg/m^2^)	Below P75	27.31 ± 5.68	< 0.01^b^
	Above P75	32.88 ± 5.35	

^a^P-value obtained through Mann-Whitney test; ^b^P-value obtained through independent t test.

LAP = lipid accumulation product; SD = standard deviation; HDL = high-density lipoprotein; LDL = low-density lipoprotein; P75 = percentile 75.

## DISCUSSION

Although most guidelines recommend a mixture of screening measures to identify people who are at relatively high risk of CVD, the pre-existing tools are not particularly effective for reducing the risk of cardiovascular events.^13^ The results from the present study showed that the LAP index presented good correlations with the metabolic profile and anthropometric variables. This emphasizes the hypothesis that it is an easy-to-use and practical tool for detecting interactions between excess adiposity and cardiovascular risk. It may therefore be useful within primary healthcare services that face financial constraints that hinder access to cardiovascular risk markers of greater sophistication.

Many cardiovascular risk assessment systems are available for use among apparently healthy individuals, including Framingham,^16^ SCORE,^17^ CUORE^18^ and others. In practice, most risk estimation systems perform rather similarly when applied to populations that are recognizably comparable to those from which the risk estimation system was derived. However, these tools sometimes are not useful in situations in which it is necessary to ascertain a greater number of cardiovascular risk variables (including anthropometric and biochemical values), and in which the time available for assessing the individual is limited.

Thus, the LAP index is a useful tool because it only includes two variables, which are usually available cheaply in a wide variety of healthcare services. Recently, Xie et al.^19^ evaluated cardiovascular risk among Chinese patients with growth hormone deficiency, using the Framingham score and LAP index. In comparison with healthy controls, they found that the LAP index was higher among adult growth hormone deficiency patients, but that these higher LAP indexes were not associated with Framingham risk. This was probably observed because there was no common factor between the LAP index and Framingham risk score.

Only a few studies have aimed to evaluate the LAP index in different populations. Some of these studies have proposed a cutoff point using a receiver operator characteristic (ROC) curve.^6^^,^^20^^,^^21^ Wakabayashi and Daimon^6^ evaluated 10,170 Japanese workers (35-40 years old) and proposed cutoff values for the LAP index of 21.1 cm.mmol/l and 37.2 cm.mmol/l for women and men, respectively. These values were lower than those observed in the present study because our sample had a higher mean age (48.4 years) and was stratified into nutritional status categories (overweight or not overweight). However, our results also showed that women had lower LAP index values than those of men. Nascimento et al.^21^ found a high cutoff point through using an ROC curve in a cross-sectional study conducted on 78 women aged 18 to 42 years who presented polycystic ovarian syndrome and were attended at a university hospital in Brazil. Except for HDL levels, logistic regression showed that all cardiovascular risk markers presented a higher chance of being altered when the LAP index was above the cutoff value of 37.9 cm.mmol/l. This higher value may have been found because women with polycystic ovarian syndrome already present higher cardiovascular risk than that of the general population. Lastly, in a regional hospital in southern Taiwan, Chiang and Koo^20^ evaluated 513 individuals and showed that the optimal cutoff for the LAP index was 28.4 ­cm.­mmol/l, for both sexes.

Our results showed a difference in the distribution of the LAP index between people who were overweight and those who were not overweight, thus showing that the index had good discriminatory power regarding cardiovascular risk. Other studies have also shown a correlation between higher LAP values and the risk of cardiovascular events in clinical populations.^19^^,^^20^^,^^21^ This has also been observed in some subgroups of normal-weight subjects who were metabolically obese.^9^

In a longitudinal study, Du et al.^22^ evaluated 3,552 subjects with normal weight and showed that through using an ROC curve, the LAP index was more effective than were anthropometric parameters for identifying normal and metabolically obese individuals, thus demonstrating the power of the LAP index for determining the presence of cardiovascular risk.

In another study, Xia et al.^23^ evaluated a representative sample of 2,524 Chinese non-diabetic individuals. Comparison with the results from using BMI and WC showed that use of the LAP index had a greater impact on the insulin resistance index. Moreover, in multivariate analysis, the LAP index had a greater impact on homeostatic model assessment (HOMA) than did BMI and WC. These results showed that the LAP index really discriminated among biochemical markers, regardless of general adiposity.

Some limitations of our study should be considered in interpreting the results. We did not evaluate insulin resistance and we did not have any medical diagnosis for diabetes or other cardiovascular diseases among our cases. Furthermore, we did not use any imaging technique to detect body compartments, especially with regard to overall and visceral fat, to correlate with all the anthropometric indexes. Thus, it was not possible suggest any cutoff point for screening for these diseases through analyzing an ROC curve, for example. We suggest that future studies should evaluate insulin resistance or diabetes in such patients, given that the association between cardiovascular risk and diabetes is well-established.

### CONCLUSION

Through using the LAP index, a notable number of people presenting cardiovascular risk factors could be identified among primary healthcare users in Brazil. High cardiovascular risk according to the LAP index seemed to be associated with anthropometric and biochemical cardiovascular risk markers. These significant correlations between the LAP index and the traditional biochemical risk markers may be useful within conventional clinical practice, for cardiovascular risk screening in primary healthcare.
